# Schizophrenia and Hereditary Polyneuropathy: *PMP22* Deletion as a Common Pathophysiological Link?

**DOI:** 10.3389/fpsyt.2019.00270

**Published:** 2019-05-02

**Authors:** Dominique Endres, Simon J. Maier, Christiane Ziegler, Kathrin Nickel, Anne N. Riering, Benjamin Berger, Johann Lambeck, Miriam Fritz, Birgitta Gläser, Friedrich Stock, Michael Dacko, Thomas Lange, Irina Mader, Katharina Domschke, Ludger Tebartz van Elst

**Affiliations:** ^1^Section for Experimental Neuropsychiatry, Department of Psychiatry and Psychotherapy, Medical Center—University of Freiburg, Faculty of Medicine, University of Freiburg, Freiburg, Germany; ^2^Department of Psychiatry and Psychotherapy, Medical Center—University of Freiburg, Faculty of Medicine, University of Freiburg, Freiburg, Germany; ^3^Department of Neurology, Medical Center—University of Freiburg, Faculty of Medicine, University of Freiburg, Freiburg, Germany; ^4^Institute of Human Genetics, Medical Center—University of Freiburg, Faculty of Medicine, University of Freiburg, Freiburg, Germany; ^5^Institute of Human Genetics, University Hospital Münster, Münster, Germany; ^6^Department of Radiology, Medical Physics, Medical Center—University of Freiburg, Faculty of Medicine, University of Freiburg, Freiburg, Germany; ^7^Department of Neuroradiology, Medical Center—University of Freiburg, Faculty of Medicine, University of Freiburg, Freiburg, Germany; ^8^Department of Radiology, Clinic Schön Vogtareuth, Vogtareuth, Germany

**Keywords:** hereditary polyneuropathy, schizophrenia, psychosis, PMP22, hereditary neuropathy with liability to pressure palsy

## Abstract

**Background:** Schizophrenic disorders are common and debilitating due to their symptoms, which can include delusions, hallucinations, and other negative symptoms. Organic forms can result from various cerebral disorders. In this paper, we discuss a potential association between schizophrenia and hereditary polyneuropathies (PNPs).

**Case presentation:** We present the case of a 55-year-old female patient with chronically paranoid–hallucinatory schizophrenia, severe cognitive deficits since the age of 30, and comorbid repeated focal pressure neuropathies beginning at age 20. At the age of 35, genetic testing revealed a deletion on chromosome 17p12 covering the peripheral myelin protein 22 gene (*PMP22*), which led to the diagnosis of hereditary neuropathy with liability to pressure palsy (HNPP). Cerebral magnetic resonance imaging showed internal atrophy, magnetic resonance spectroscopy found alteration of the glutamate and myo-inositol levels in the anterior cingulate cortex, neuropsychological testing showed deficits in working memory and psychomotor speed, and electrophysiological testing detected signs of sensorimotor demyelinating PNP (accentuated in the legs).

**Conclusion:** There may be an association between schizophrenia and HNPP. In observational studies, the deletion of interest (chromosome 17p12) was nearly 10 times more common in schizophreniform patients than in controls. This potential association could be pathophysiologically explained by the role of PMP22, which is mainly expressed in the peripheral nervous system. However, *PMP22* mRNA and protein can also be found in the brain. PMP22 seems to play an important role in regulating cell growth and myelination, functions that are disturbed in schizophrenia. Such a connection obviously cannot be clarified on the basis of one case. Future studies should analyze whether patients with HNPP exhibit increased rates of psychotic disorders, and patients with schizophrenia and repeated focal pressure neuropathies should be examined for the *PMP22* mutation. Alternatively, the co-occurrence of schizophrenia and HNPP could be coincidental.

## Background

Schizophrenia is a common disorder with a prevalence rate of about 1% (1). The clinical presentation is characterized by hallucinations, delusions, loss of self-boundaries, disorganized thinking and speech, cognitive deficits, lack of motivation, and social withdrawal (1). Secondary, organic forms can result from various cerebral disorders that are caused by genetic (22q11 deletion syndrome, cerebrotendinous xanthomatosis, Niemann–Pick type C, etc.), immunological (limbic encephalitis, anti-NMDA-R encephalitis, Hashimoto encephalopathy, etc.), infectious (neuroborreliosis, neurosyphilis, etc.), epileptic (paraepileptic psychosis, etc.), traumatic (traumatic brain injury), or neurodegenerative (frontotemporal dementia, etc). factors (2, 3).

The familial aggregation of schizophrenia is well established (1, 4). A variety of single-nucleotide polymorphisms (SNPs) in hundreds to thousands of genes have been implicated in the pathophysiology of the endogenous variants of schizophrenia (5). In these complex genetic forms, single genes only have a small effect size, and environmental factors are further important modulators. In contrast, secondary genetic forms are either monogenetic or oligogenetic: here, only one or a small number of genetic variants are important for the expression of clinical characteristics, such as copy number variants (CNVs), which are defined by deletions, duplications, or insertions of deoxyribonucleic acid (DNA) fragments, and chromosome aberrations (6, 7). In these forms, genetic variation in single genes has a high effect size, and environmental factors are considered to be less important (3). Secondary monogenic or oligogenic forms may potentially also occur in the context of hereditary polyneuropathies (hPNPs) (8). However, the association between schizophrenia and hPNPs is largely unknown. The hPNPs include isolated hPNPs (hereditary sensory neuropathy, hereditary sensory and autonomic neuropathy, and hereditary neuropathy with liability to pressure palsy or HNPP) and hPNPs in the context of systemic disorders (e.g., acute intermittent porphyria and Fabry’s disease). These systemic disorders are characterized by a monogenic or oligogenic background similar to that of secondary schizophrenia.

Searching PubMed for “hereditary polyneuropathy AND (schizophrenia OR psychosis)” yielded only eight results (as of 23 December 2018). One observational study discussed an association between hereditary spastic paraparesis and schizophrenia (8). In addition, an association between hereditary spastic paraplegia and psychosis in a female patient due to dysmorphic changes in her corpus callosum has been described independently (9). No results were returned when the same literature research was performed using “HNPP AND (schizophrenia OR psychosis)”. A nonsystematic literature search also showed that there might be a link between transthyretin-associated polyneuropathy (PNP) and schizophrenia or depression (10). Charcot–Marie–Tooth disorder, which is caused by a duplication of the *PMP22* gene, and coincident psychosis were reported in monozygotic twins (11, 12).

## Case presentation

We present the case of a 55-year-old female Caucasian patient trained as an occupational therapist who has suffered from chronic paranoid–hallucinatory schizophrenia since the age of 30. She continuously showed positive symptoms with superimposed exacerbations. At the age of 34, she was forced to retire early from her career due to her illness. Her delusions included the idea that she had sinned and needed to die, and she perceived diverse signs as confirmation of these delusions. She suffered from auditory hallucinations (voices from God, the devil, and her dead partner or mother), visual hallucinations (visions of angels), and a loss of self-boundaries (believing that other people could read her thoughts). Negative symptoms included a lack of motivation, flattened mood, and social withdrawal. Cognitive impairment has been observed since the onset of psychotic symptoms, with inattention and increasing deficits in working memory.

Intermittently, the patient abused alcohol (at least four beers per day) and benzodiazepines, but no illegal drugs. Her consumption of these substances increased during psychotic exacerbations with social withdrawal. The early death of her life partner reinforced this withdrawal. Since the onset of the disease, she had attempted suicide 10 times. Therefore, there were frequent inpatient stays in different psychiatric hospitals. Neither various neuroleptic treatments with average or high doses of aripiprazole, amisulpride, clozapine, haloperidol, perazine, pimozide, quetiapine, and risperidone, nor anticonvulsive treatment with valproate as an augmentation strategy led to full remission. Under different combination treatments, the described symptoms persisted at a reduced level.

At age 20, the patient developed clinical signs of HNPP. Initially, she was quickly fatigued and showed transient hypoesthesia of the left arm and foot. She developed transient foot dorsi-flexor paresis twice on the right side. The symptoms occurred after mechanical pressure on the corresponding body regions. In the further course of the disease, she developed transient left brachial plexus paresis at the age of 32 and again at the age of 35. When she was 35, genetic testing revealed a deletion on chromosome 17p12 involving the peripheral myelin protein 22 gene (*PMP22*), confirming the diagnosis of HNPP. The most recent neurological examination showed a discrete foot dorsi-flexor paresis on the right side (Medical Research Council score M4), absent Achilles tendon reflexes on both sides, slight pallhypesthesia of the lower extremity accentuated on the right side (5/8 on the right versus 6/8 on the left), and an ataxic and unsteady gait.

### Developmental, Somatic, and Family History

The patient’s developmental history was negative for *in utero* or birth complications, febrile convulsions, inflammatory brain diseases, and cerebral contusions. There was no evidence of any neurodevelopmental disorder. In the first two decades, the patient’s premorbid personality showed dependent traits. Her somatic medical history included only hypothyroidism, which was diagnosed at age 43. Her family history was positive for schizophrenia and HNPP. Her father’s half-brother suffered from schizophrenia. The patient’s older brother (transient hypesthesia of parts of one hand; side and explicit location unclear) and father (paresis of the shoulder abductors) potentially also suffered from HNPP (no genetic diagnostics performed, clinical reports not available). Her younger brother and her mother were healthy.

### Basic Investigations and Magnetic Resonance Spectroscopy

**Figure 1:**
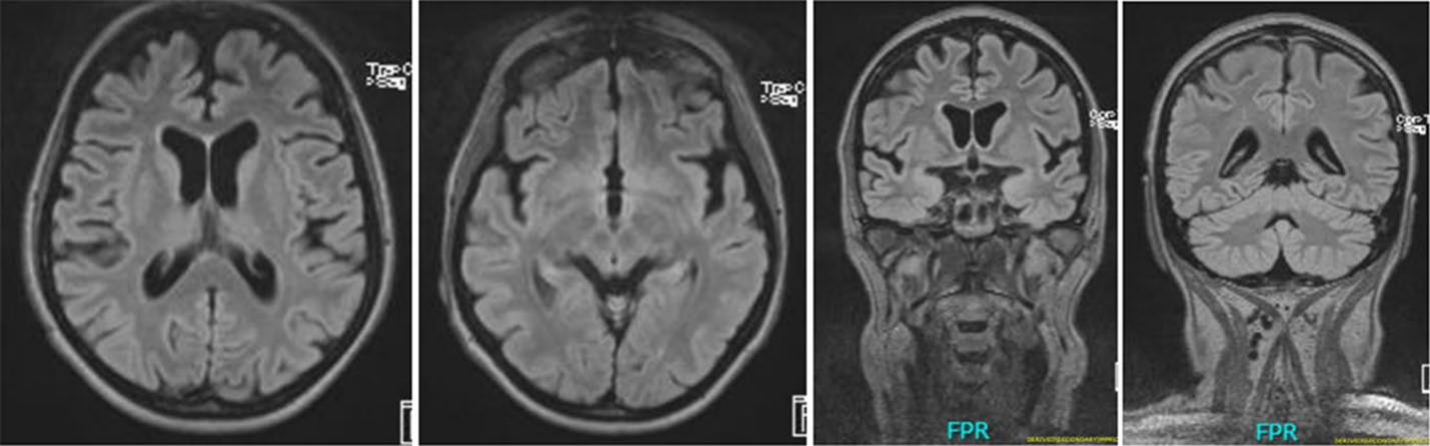
Cerebral magnetic resonance (cMRI) imaging showing generalized atrophy with perisylvian accentuation.

**Figure 2:**
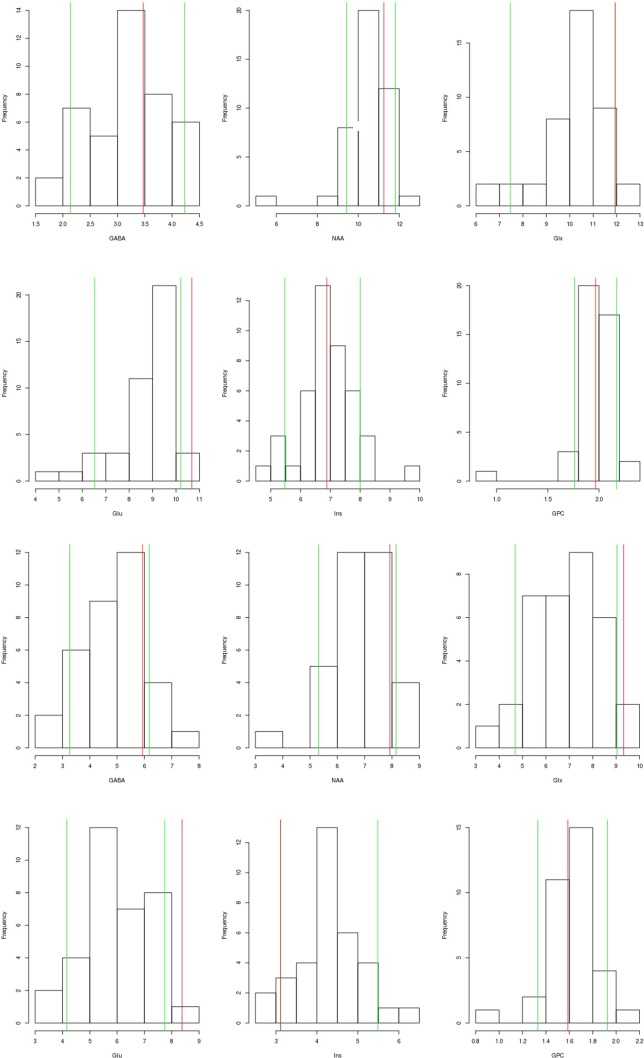
Magnetic resonance spectroscopy (MRS) findings: We present the findings of the dorsal anterior cingulate cortex (dACC; the two upper rows) and the pregenual anterior cingulate cortex (pACC; lower rows). The patient’s values (red line) are compared to the values of a healthy control group measured in another study (N = 43, mean age: 35, gender ratio: 14 females/29 males). The green line shows the 90% reference interval of the control group. For Glx in the dACC and Ins in the pACC the green (90% reference interval) and the red line (patient’s values) are superimposed, therefore only the red line is visible. The measured metabolite concentrations have been corrected for MRS voxel composition (content of gray matter, white matter, and cerebrospinal fluid), as well as for the influence of age and differences in the signal-to-noise ratio of the MRS measurements. Abbreviations: GABA, gamma-aminobutyric acid including coedited macromolecules; NAA, N-acetylaspartate; Glx, glutamate and glutamine; Glu, glutamate; Ins, myo-inositol; GPC, glycerophosphorylcholine.

The serum, cerebrospinal fluid, and cerebral magnetic resonance imaging (cMRI) investigations did not show any evidence of an immunological cause for schizophrenia or PNPs. The electrophysiological tests revealed sensorimotor, demyelinating PNP (accentuated in the legs). Neurosonography showed bilateral enlargement of the median and ulnar nerve at typical sides of entrapment syndromes. Neuropsychological testing showed considerable deficits in working memory and a mild deficit in psychomotor speed. The electroencephalography (EEG) showed rare intermittent slowing. The cMRI showed generalized atrophy (Figure 1). Magnetic resonance spectroscopy (MRS) was performed in the dorsolateral prefrontal cortex (DLPFC), the dorsal anterior cingulate cortex (dACC), and the pregenual anterior cingulate cortex (pACC) using a MEGA PRESS sequence (repetition time = 1,500 ms, echo time = 68 ms, flip angle = 90°) and the acquired spectra were quantified with the software LCModel. Glutamate concentrations in the dACC and pACC were relatively high and outside the 90% reference intervals. The myo-inositol concentrations in the pACC were relatively low and within the 90% reference interval (Figure 2). The DLPFC measurement displayed poor spectral quality and was therefore not analyzed. The diagnostic findings are summarized in Table 1.

**Table 1:** Current diagnostic findings.

**Serum analyses**	Blood cell count, electrolytes, liver/kidney values, HbA1c, cobalamin, folic acid, and immunofixation were normal. Thyroid-stimulating hormone (TSH), triiodothyronine, and thyroxine levels were in normal ranges. Autoantibodies against thyroglobulin and thyroid peroxidase and against TSH receptor were not increased. Screening for infections (borreliosis, lues, and HIV) was negative. No antibodies against intracellular onconeural antigens (Yo, Hu, CV2/CRMP5, Ri, Ma1/2, SOX1) or intracellular synaptic antigens (GAD, amphiphysin) were found. Screening for antinuclear antibodies (ANA), anti-neutrophil cytoplasmic antibodies (ANCA), antiphospholipid antibodies (APA), and rheumatoid factor (RF) was negative.	
**Cerebrospinal fluid analyses**	Normal white blood cell count (1/µl; reference < 5/µl). Increased protein concentration (615 mg/L; reference < 450 mg/L), but normal age-corrected albumin quotient: 7; age-dependent reference < 8 × 10^−3^). No CSF-specific oligoclonal bands; IgG Index not increase (0.45; reference ≤ 0.7). Antibodies against neuronal cell surface antigens (*NMDAR, AMPA-R, GABA-B-R, VGKC-complex [LGI1, Caspr2]*) were negative.	
**Cerebral magnetic resonance imaging**	Slight generalized atrophy with perisylvian accentuation. Very mild microangipathic white matter lesions.	
**Magnetic resonance spectroscopy**	dACC: High glutamate concentrations. pACC: High glutamate and low myo-inositol concentrations. DLPFC: Not analyzed due to bad spectral quality.	
**Electroencephalography** (including a hyperventilation episode)	Alpha-rhythm, rare intermittent slowing, no epileptiform activity.	
**Electrophysiological measurements**	Visual evoked potentials: normal. Motor nerve conduction study (NCS): Right ulnar nerve: increased distal motor latency, borderline distal nerve conduction velocity, significant reduction of the nerve conduction velocity in the cubital tunnel, F-waves not reproducible. Right tibial nerve: increased distal motor latency, increased F-wave latency. Left peroneal nerve: distal CMAP strongly reduced, proximal CMAP not reproducible, so nerve conduction velocity not measurable. Sensory NCS: Left radial nerve (superficial ramus): normal amplitude, reduced nerve conduction velocity. Left sural nerve: sNAP not reproducible.	
**Neurosonography**	Median nerve bilaterally enlarged in carpal tunnel (CSA carpal tunnel 19–21 mm^2^, CSA forearm 7 mm^2^, wrist-to-forearm ratio 2.7–3). Ulnar nerve bilaterally enlarged in cubital tunnel (CSA cubital tunnel 12–17 mm², CSA upper arm 6–7 mm², humerus-to-elbow ratio 1.7–2.8).	
**Genetic testing**	MLPA analysis: heterozygous deletion of the *PMP22* gene. Karyotyping: normal female karyotype 46,XX. DNA microarray (array CGH): heterozygous microdeletion on chromosome 17p12 (1.33 Mb—125 contiguous oligonucleotides)	
Neuropsychological tests and *z* values*	Working memory (digit span—WAIS-IV) Verbal learning (VLMT) Verbal memory (VLMT) Working speed (digit symbol—WAIS-V) Phonematic fluency (RWT) Semantic fluency (RWT)	−2.0** −0.9 −0.1 −1.0** 0.4 −0.7

CSA, cross-sectional area

*Neuropsychological assessment was interrupted repeatedly due to the patient’s spontaneous delusional utterances and experiences. This might reduce the validity of test results.

**Below average.

### Genetic Investigations: Cytogenetic and Array Analysis

MLPA (multiplex ligation-dependent probe amplification) analysis revealed a heterozygous deletion of the *PMP22* gene on chromosome 17p12. To delineate the extent of this deletion, we additionally performed conventional karyotyping and array CGH (comparative genomic hybridization).

Conventional R-banded karyotypes from the patient were analyzed according to standard protocols with a resolution of approximately 500 bphs and revealed a structurally and numerically normal female karyotype (46,XX) in all 28 metaphases examined. Furthermore, the genomic DNA of the patient was examined by microarray analysis (CytoSureTM constitutional v3 array 180k; Oxford Gene Technology) according to the manufacturer’s instructions. After hybridization, the array was scanned with the SureScan microarray scanner (Agilent); the results were analyzed using CytoSure interpret software v.4.9 (Oxford Gene Technology) and the Genome Reference Consortium human genome GRCh37 (hg19). Molecular karyotyping revealed a heterozygous deletion of approximately 1.33 Mb (125 contiguous oligonucleotides) out of the chromosomal region 17p12 (karyotype after the International System for Human Cytogenetic Nomenclature, 2016: arr[GRCh37] 17p12(14111972_15442257)x1). The deletion encompasses i.a. the genes *COX10*, *HS3ST3B1*, *PMP22*, *TEKT2*, and *CDRT4*. A deletion of that extent in 17p12 is found in nearly 80% of patients with HNPP (13). Further chromosomal deletions or duplications that might have etiologically contributed to our patient’s disease were not detected.

### Differential Diagnosis

The patient’s psychiatric symptoms were compatible with paranoid–hallucinatory schizophrenia (ICD-10: F20.0). The PNPs in combination with the deletion of the *PMP22* gene led to the diagnosis of HNPP (ICD-10: G60.0). Due to the potential secondary schizophrenia in the context of HNPP, a psychotic disorder with delusions due to a known physiological condition might ultimately be diagnosed (ICD-10: F06.2).

## Discussion

In this paper, we present the case of a female patient with chronic paranoid–hallucinatory schizophrenia with poor response to therapy and comorbid HNPP.

### HNPP, *PMP22* Deletion, and Central Nervous System Involvement

HNPP is an autosomal-dominant, peripheral neuropathy characterized by repeated and transient episodes of focal pressure neuropathies at compression-exposed sites (e.g., brachial plexus, sciatic nerve) (14). Our patient suffered from repeated foot flexor paresis and twice experienced brachial plexus paresis. In HNPP patients, nerve conduction study (NCS) often reveals demyelinating PNP with nerve suffering located predominantly in entrapment sites (15). In our patient, NCS showed findings compatible with sensorimotor, demyelinating PNPs with an entrapment syndrome in the right cubital tunnel. Histologically, sural nerve biopsies of HNPP patients typically display tomaculae, which are redundantly overfolded layers in the myelin coat (16). Genetically, a 1.5-Mb deletion on chromosome 17p12, including the *PMP22* gene, is found in most cases (17). Molecular karyotyping in our patient showed a heterozygous deletion of 1.33 Mb.

The *PMP22* gene is mainly expressed in the peripheral nervous system (PNS); however, mRNA and protein can also be found in the central nervous system (CNS) (18). Recent studies suggested the important involvement of the CNS in most patients with *PMP22* deletion (18, 19). This is supported by prolonged latencies of visual evoked potentials, neurochemical alterations with decreased *N*-acetylaspartate (NAA) and creatine (Cre) concentrations, white matter (WM) volume reduction detected by cMRI, cognitive impairment (in 70% of patients), and fractional anisotropy alteration in several WM regions (e.g., in the columns of the fornix) (18–20). A large family study described CNS involvement and WM lesions predominantly in the subcortical frontal WM (21). Therefore, in line with the studies described, we hypothesize****that our patient’s schizophreniform symptoms may represent CNS involvement. This consideration is clinically supported by an insufficient therapy response to neuroleptics, severe cognitive deficits, EEG slowing, internal brain atrophy, and neurometabolic alterations (high glutamate in the dACC and pACC and low myo-inositol in the pACC). Compared with the patient group from Chanson and colleagues, we also found brain atrophy and neuropsychological deficits; however, in the presented case, NAA concentration and visual evoked potentials were normal, and DTI measurements were not performed (18). A causal relationship between the poor response to therapy and the *PMP22* deletion remains speculative and is not proven by the case report. However, our hypothesis is also supported by several epidemiological and pathophysiological ideas, which we discuss in detail in the following paragraphs along with potential limitations.

### *PMP22* Deletion in Patients With Schizophrenia

Copy number variation in the chromosomal region 17p12 has repeatedly been implicated in schizophrenia (22–25). In a study analyzing the involvement of rare CNVs in 471 patients with schizophrenia, a deletion on 17p12 was found in two patients but not in controls. Therefore, the authors reanalyzed the data from two recent, large CNV studies of schizophrenia and found a *PMP22* deletion in 6 out of 4,618 (0.13%) patients and 6 out of 36,092 (0.017%) controls (26–28). The data demonstrate that the described 17p12 deletion can be found nearly 10 times more often in schizophrenic patients compared to healthy controls (28). Moreover, there is at least one case report presenting a patient with schizophrenia in combination with mental retardation and *PMP22* deletion without hPNPs (29).

### Pathophysiological Considerations

PMP22 is a small, hydrophobic membrane glycoprotein that is mostly expressed by Schwann cells and comprises 2–5% of PNS myelin proteins in humans (16, 30). *PMP22* mRNA and protein were also detected in the CNS, specifically in most parts of the brain (especially the corpus callosum) and the spinal cord. Changes in *PMP22* mRNA may explain myelin abnormalities because it plays a role in the regulation of cell growth (even in the absence of protein), which was first described in fibroblasts (30, 31). Reductions in *PMP22* mRNA have been observed in the hippocampus and anterior cingulate cortex in the postmortem brains of patients with schizophrenia (32). The PMP22 protein was restricted to a few areas [anterior horn and pia mater of the spinal cord, preganglionic sympathetic neurons; (33)]. In summary, *PMP22* mRNA and protein are important for the regulation of cell growth and the maintenance of myelin integrity and therefore ensure the propagation of action potential (16). Heterozygous deletion of the *PMP22* gene results in a loss-of-function phenotype (16) and altered myelination, as found in patients with schizophrenia (34).

### Clinical Importance of Case Studies and Limitations

It is important to note that the co-occurrence of schizophrenia and HNPP could be coincidental, which is supported by the absence of schizophreniform symptoms in our patient’s brother and father, who both very likely suffered from HNPP. However, genetic analyses of the patient’s brother and father were not performed to our knowledge. So, we can only speculate whether the discrepancy in psychiatric symptoms between our patient and her brother and father could be due to a variable expression and an incomplete penetrance, respectively, of the genetic effect of the *PMP22* deletion. Besides environmental factors, further genetic variants might have contributed to our patient’s disease, particularly since the presently identified chromosomal deletion additionally encompasses *COX10*, *HS3ST3B1*, *TEKT2*, and *CDRT4*. However, the detected radiological and neuropsychological findings can also be found in schizophrenia; therefore, they are not clearly associated with the PMP22 deletion (34).

Case studies reporting such potential associations are essential to inspire further clinical trials (35). Retrospective studies analyzing psychiatric comorbidity in patients with HNPP could demonstrate whether there is a relevant association between the disorders and confirm or refute the pathophysiological role of *PMP22* deletion in a subgroup of patients with schizophrenia.

## Conclusion

There may be an association between schizophrenia and HNPP, which could be explained by the role of *PMP22* in regulating cell growth and myelination. If such an association existed, which, of course, cannot be clarified from one case, then it might explain the poor control of psychiatric symptoms in our patient. But *PMP22* deletion does not necessarily mean causality for the emergence of psychotic symptoms. For further clarification, patients with psychotic disorders and repeated transient focal neurological symptoms (which could be explained by pressure neuropathies) should be examined for *PMP22* gene variation. Future studies should analyze whether patients with HNPP exhibit increased rates of schizophrenia. Alternatively, the co-occurrence of schizophrenia and HNPP could be coincidental.

## Ethics Statement

The patient has given her signed, written informed consent for this case report, including the images presented, to be published.

## Author Contributions

DE and LT treated the patient. DE performed the data research and wrote the paper. SM, KN, MD, TL, and IM performed and interpreted the magnetic resonance spectroscopy. FS and BG performed the genetic testing. CZ and KD supported the interpretation of genetic findings. BB, JL, and MF performed and interpreted the electrophysiological measurements, neurosonography, neurological examination, and CSF measurements. IM performed and inter­preted the cMRI. AR performed the neuropsychological testing. All authors were critically involved in the theoretical discussion and composition of the manuscript. All authors read and approved the final version of the manuscript.

## Funding

The article processing charge was funded by the German Research Foundation (DFG) and the University of Freiburg in the funding program Open Access Publishing.

## Conflicts of Interest Statement

BB received travel grants and/or training expenses from Bayer Vital GmbH, Ipsen Pharma GmbH, Norvartis, Biogen GmbH, and Genzyme, as well as lecture fees from Ipsen Pharma GmbH, Alexion Pharma GmbH, Merck, Sanofi Genzyme, and Roche. IM received lecture fees from UCB Pharma GmbH, Germany. LTvE received advisory boards, lectures, or travel grants within the last 3 years: Eli Lilly, Janssen-Cilag, Novartis, Shire, UCB, GSK, Servier, Janssen, and Cyberonics.

The remaining authors declare that the research was conducted in the absence of any commercial or financial relationships that could be construed as a potential conflict of interest.
